# Effect evaluation of kangaroo mother care in Liping area, Guizhou province,China

**DOI:** 10.1186/s12887-022-03723-2

**Published:** 2022-11-08

**Authors:** Wu Li, Zhao Yu, Yang Jing

**Affiliations:** 1 Chongqing Health Center For Women And Children, Chongqing, 400012 China; 2 Maternal and Child Health Hospital of Liping County, Guizhou Province, Guizhou, 557300 China; 3 Guizhou Provincial People’s Hospital, Guizhou, 557300 China

**Keywords:** Kangaroo mother care, Health and poverty alleviation, Western region, Mother and child safety, Breastfeeding

## Abstract

**Backgroud:**

Kangaroo mother care (KMC) refers to the mother and baby after the birth of the early start of continuous skin contact way of a newborn care, which is a simple operation, easy controlled and with low cost, no large or high consumption of equipment.So it is very suitable for developing in areas where medical resources are relatively scarce, such as GuiZhou province where is a relatively poor province in China with many ethnic minorities.

**Methods:**

This study selected the pregnant women who gave birth in Liping County, Guizhou Province, China, as the research object, to explore the impact of kangaroo mother care on the physiologic status of newborns in liping county, Guizhou Province.

**Results:**

A total of 347 hospitalized parturient women were divided into the KMC group and the control group. The results showed that the KMC group showed obvious advantages in stabilizing newborn vital signs, health indicators, promoting the success rate of breastfeeding and reducing newborn pain.

**Conclusions:**

Research shows that kangaroo mother care is beneficial to postpartum maternal and infant health, and has advantages suitable for local characteristics, which is worth further promotion in minority areas of Guizhou Province.

## Backgroud

China is located in the east of Asia and on the west coast of the Pacific, with a total population of 1,411,178 million [1]**.** There are 34 provincial-level administrative regions in China (including 23 provinces, 5 autonomous regions, 4 municipalities directly under the Central Government and 2 special administrative regions). As one of the 34 provincial administrative regions in China, Guizhou province is located in southwest China. The permanent resident population of Guizhou province is 38.562 million, among which the minority population accounts for 36.4% [2]^**.**^ It has the largest Miao and Dong tribes in the world, and is one of the provinces with the largest minority population proportion and the largest variety in China. In 2020, the rate of prenatal check-up, postnatal visit, hospital delivery, infant mortality and neonatal mortality in Guizhou province was 96.63%, 94.22%, 99.63%, 5.01‰ and 2.61‰ respectively [2].

Postpartum mother-infant skin contact is the core content of the WHO/China CDC neonatal safety project. It refers to the process of continuous mother-infant skin contact and frequent sucking of the mother's nipple to achieve full breastfeeding early after delivery. In the previous research of this research group, it has been confirmed [3] that extending the postpartum mother-to-child skin contact time has a positive impact on enhancing breastfeeding confidence, increasing the rate of exclusive breastfeeding, and achieving long-term breastfeeding. The further deepening of development in Guizhou has laid a foundation for practice. Kangaroo mother care (KMC) is a continuation of the preliminary work of this project. It refers to the continuous skin contact between the mother and the newborn at an early stage, frequent sucking of the mother’s nipples, to achieve full breastfeeding, and can continue to be implemented for a long time after delivery A kind of newborn care method [4], which can effectively prevent newborns, especially premature infants, from being exposed to various adverse stimuli from the outside [5]. Liping County, Guizhou Province, as a pilot county for the neonatal safety project, took the lead in developing KMC in Guizhou minority areas in 2018. This study conducted relevant data analysis on women and newborns participating in kangaroo care in this area. The maintenance of vital signs of newborns, newborn health indicators (newborn sleep, newborn feeding status, newborn mood), breastfeeding and newborn pain are evaluated in various aspects, aiming to explore a way to promote the growth and development of newborns and meet the development of the region The level of maternal and child health care escorts the health of women and children.

## Methods

### Objects

Participants In this study, newborns and their mothers who were hospitalized in the obstetrics of Liping County Maternity and Child Health Hospital between December 2019 and December 2020 were selected. According to the wishes of the patients, mothers and newborns who voluntarily participated in KMC were selected as the KMC group, a total of 186 cases. In the traditional way, newborn infants were placed in a stroller and followed by medical care as a control group, with a total of 161 cases. The KMC group performed kangaroo care in the KMC ward set up in the obstetric ward.

Inclusion criteria: 1) Accept KMC on a voluntary basis, and the parturient and family members sign informed consent; 2) The parturient or other major KMC implementers have no history of major illnesses; 3) Have certain learning ability and normal communication skills; 5) Mother Babies are generally in good condition, with stable vital signs; 6) Late preterm infants (gestational age between 34–36 weeks [+ 6]), preterm infants with stable vital signs; 7) Term low birth weight infants with stable vital signs; 8) Mother Newborns with obstetric comorbidities and complications and the mother's postpartum condition is stable. Exclusion criteria: 1) Newborns whose vital signs are not stable; 2) KMC implementers suffer from infectious diseases that may increase the risk of infection through skin contact and the mother’s own conditions that are not suitable for breastfeeding; 3) Newborns who require close medical observation; 4) Birth weight is less than 2000 g; 5) Newborns with diseases that are not suitable for KMC. Halfway withdrawal criteria: 1) The parturient has severe malaise, fever, nausea, vomiting, diarrhea, etc.; 2) The parturient has bad emotions and is unwilling to continue KMC or breastfeeding; 3) The parturient has a situation that requires treatment; 4) The newborn has a condition unsteady vital signs; 6) sudden accidents of newborns; 7) newborns with diseases that affect breastfeeding.

### Common intervention content

Joint intervention content 1) Propagating and educating pregnant women in obstetric outpatient clinics through multimedia broadcasting, setting up publicity boards, and distributing brochures; 2) Conducting centralized educating and educating pregnant women and their families in hospital through slide interpretation + oral education; 3) Voluntary participation KMC pregnant women and their family members sign informed consent; 4) KMC operation and precautions are trained for pregnant women and their families participating in KMC; 5) During the implementation of KMC, the tube bed nurse collects and reports data, and the main members of the project team Data collection and analysis, and regular quality control.

Mothers are encouraged to perform KMC as soon as possible after childbirth. The nurse can adjust the indoor temperature and humidity in the KMC implementation area according to the guideline of KMC operation [6]. They change diapers, wear socks and hats, and place the newborn in front of the mother's bare chest and abdomen,in order to increase the contact area between the newborn and the mother.Instruct the mother to place one hand on the baby's back and one hand to hold the buttocks to avoid slipping. Do not limit the time of kangaroo-style care and encourage continuous and long-term skin contact. The main participant is the mother of the newborn, or relatives such as the father or grandmother. If the mother interrupts KMC due to physical factors or no other family members participate Next, perform KMC every morning, noon, and evening, and each skin contact time should be no less than 1 h.

### Observation indicators

Neonatal vital signs monitoring Record the four body temperature and breathing of newborns in the KMC group and the control group during the implementation of KMC.

The evaluation method of neonatal pain is by changing diapers or collecting heel blood. Within a short period of time (not more than 1 min) after the stimulation occurs, the newborns in the KMC group are carried back to the mother’s chest for KMC, and the newborns in the control group undergo KMC. Embrace and comfort. After the newborn is carried back, observe and record the newborn's performance according to the scale indicators. This study refers to the Neonatal Infant Acute Pain Assessment Scale [7] (Neonatal Infant Acute Pain Assessment Scale, NIAPAS), which is a professional tool for assessing pain from multiple dimensions, including gestational age, alertness, 9 indicators such as response to operation, heart rate, respiration, etc., with a total score of 0–18. The higher the total score, the more severe the pain. The internal consistency reliability of the scale Cronbach's α coefficient is 0.723, the inter-rater reliability is 0.991 ~ 0.997, and the reliability is relatively high [[4]]. Because the scale is relatively objective, it can be better used for both full-term infants and premature babies.. In the evaluation of pain in premature infants, this study selected them for the evaluation of pain in neonates.

Comparison of newborn health indicators Observe the sleep, mood and vomiting of newborns in the KMC group and the control group within 24 h.

The newborn sleep evaluation method records the continuous sleep time of newborns in the KMC group and the control group in a quiet state, and calculates the total continuous sleep time within 24 h. The quiet state is when the baby's facial muscles are relaxed, the eyes are closed, and the breathing is even. Except for almost no startle and slight mouth movement, there is no other activity, and the baby is completely at rest [8].

Feeding condition of the newborns A few minutes after the newborns are fed and burped, the newborns in the KMC group are carried back to KMC, and the newborns in the control group are brought back to the stroller and lie on their sides. Observe the vomiting of the newborns in the two groups and record them within 24 h Number of vomiting.

The neonates’emotions were evaluated by the nursing staff, and after excluding the triggers such as hunger, after stooling, tight clothing, disease, etc., the number of daily crying and the duration of each crying of the two groups of newborns were recorded.

Breastfeeding situation This section uses the Breast-feeding Assessment Tools (BAT) developed and revised by Matthews. The measurement tool has 4 items for each latitude. The item has a minimum score of 0 and a maximum of 3 points. The total score is 0–12, and the total score is more than 8 points for successful breastfeeding [9], The reliability and validity of the scale are good, and the internal consistency coefficient of the scale in domestic research is 0.97 [10]. This part of the content is carried out by the nursing staff before the mother is discharged from the hospital. The mother will score the expression of specific items and record the specific time of successful breastfeeding of the mother with a total score of > 8 to understand the exclusive breastfeeding situation of the mother before discharge from the hospital. And implementation time.

Quality control This subject was carried out in the pilot hospital in the pilot area of ​​the neonatal safety project of the WHO/China CDC Maternal and Child Health Center (Liping County Maternity and Child Health Hospital, Qiandongnan Miao and Dong Autonomous Prefecture, Guizhou Province). It will be implemented after the review and approval of the hospital ethics committee (Li Fuyou Ji Zi [2020] No. 3). Project participants are trained and assessed in a standardized manner, and the main person in charge of the project conducts statistics and quality control on data and scales every month.

Statistical methods Use SPSS 22.0 software for statistical analysis, independent sample t test for measurement data conforming to normal distribution, single-factor analysis of variance for comparison of multiple groups of measurement data; for non-measurement data that does not conform to normal distribution The Mann–Whitney U nonparametric test was used for comparison; the RXC combined tabular chi-square test was used for the classification data. *P <* 0.05 indicates that the difference is statistically significant.

## Results

A total of 347 cases of parturients and newborns were included in this study, including 186 cases in the KMC group and 161 cases in the control group.

Comparison of the basic vital signs of newborns in the KMC group and the control group The body temperature of the newborns in the KMC group was significantly higher than that of the control group (*P <* 0.01), and the respiratory rate of the control group was not significantly different from that of the KMC (*P > *0.05), see Table 1. There was no significant difference in the frequency of four breaths between the KMC groups (*P > *0.05), and the frequency of four breaths between the control group was significantly different in at least two groups (*P <* 0.05), see Table 2.

**Table 1:** Comparison of body temperature and respiration between KMC group and control group

Groups	Number	Temperature(°C)	Breathing(Times/min)
KMC	186	36.69 ± 0.12	41.42 ± 1.14
Control	161	36.29 ± 0.20	41.51 ± 1.10
*T* value		30.89	0.76
*P* value		< 0.01	> 0.05

**Table 2:** Comparison of respiratory stability between KMC group and control group

Times (Groups)	1	2	3	4	*F* value	*p* value
KMC	41.35 ± 1.42	41.41 ± 1.53	41.31 ± 1.53	41.61 ± 1.59	1.43	0.23
Control	41.89 ± 1.98	41.40 ± 1.79	41.34 ± 1.93	41.43 ± 2.01	2.85	< 0.05

Comparison of newborn pain in the KMC group and the control group The comparison of the pain index of the newborns in the KMC group and the control group after pain stimulation is shown in Table 3. The scores of the two groups are statistically significant, *P <* 0.01.

**Table 3:** Comparison of newborn pain between the KMC group and the control group

Groups	NIAPAS Rating scale
KMC	0.56 ± 0.82
Control	1.70 ± 1.75
*t* value	7.56
*P* value	< 0.01

Comparison of newborn health indicators between the KMC group and the control group The total sleep duration of newborns in the KMC group within 24 h was longer than that of the control group, which was statistically significant (*P <* 0.01). The cumulative 24-h crying time of the newborns in the KMC group was significantly less than that of the control group ( *P <* 0.01) See Table 4. There were significant differences between the number of crying and vomiting in the KMC group and the control group (u = 12.85, *P <* 0.01, u = 4.87, *P <* 0.01).

**Table 4:** Comparison of newborn health indicators between the KMC group and the control group

Groups	Total leep duration (h)	Accumulated crying time(min)
KMC	19.73 ± 2.01	6.76 ± 7.23
Control	13.51 ± 1.70	28.53 ± 18.43
*t* value	30.89	14.07
*p* value	< 0.01	< 0.01

Comparison of breastfeeding between the KMC group and the control group. The success rate of first breastfeeding and the rate of exclusive breastfeeding before discharge from the KMC group were higher than those of the control group (*P <* 0.01). The total score of neonatal BAT in the KMC group was significantly higher than that of the control group (*P <* 0.01), the KMC group achieved the first breastfeeding success and the time of exclusive breastfeeding before discharge (compared to the birth time of the newborn) was earlier than the control group (*P <* 0.01). See Table 5.

**Table 5:** Comparison of breastfeeding status between KMC group and control group

Groups	KMC	Control	*c2/ t*	*p* value
Success rate of first breastfeeding	98.9%(184)	21.5%(35)	222.95	< 0.01
Exclusive breastfeeding rates	94.6%(176)	7.4%(12)	266.19	< 0.01
BAT score	9.18 ± 0.60	6.46 ± 1.42	22.79	< 0.01
Time of first successful breastfeeding(h)	9.87	37.50	7.56	< 0.01
Achieve exclusive breastfeeding time(h)	30.40	74.33	8.21	< 0.01

## Discussion

The impact of KMC on the basic vital signs of newborns. The main implementer of KMC is the mother of the newborn, or other family members such as the father. The skin contact provides the newborn with a suitable temperature. KMC does not require an incubator because the parents’ Body temperature is the best thermostat. When a newborn has a low body temperature, the skin of the parents can keep warm, and it can be cooled when the body temperature rises. The human skin can not only provide temperature, but also maintain a suitable humidity, which is the best for newborns. A comfortable and environmentally friendly hotbed [11]. Studies have shown that KMC can directly provide body temperature to newborns, and transmit the heartbeat and breathing of parents, it also can promote heat production in newborns, especially in late preterm infants. KMC can stabilize the central thermoregulation function of newborn [12], it also can help prevent the constriction of pulmonary blood vessels caused by hypothermia [13], at last it can reduce the occurrence of apnea, and keep breathing stable. This study shows that the average body temperature of the newborns in the KMC group is higher than that of the control group, and there is no significant difference in the average respiratory frequency. However, the respiratory frequency of the control group varies greatly, which proves that KMC helps the newborns to breathe smoothly and stabilizes vital organs., It has an active role in promoting the growth and development of newborns.

KMC has the effect of intervention on neonatal pain indicators. In this study, the KMC group and the control group did not have severe pain. The pain-free rate of the KMC group was significantly higher than that of the control group. The scores of the two groups of mild pain were analyzed, and the KMC was compared with the control group. Group is low. The main pain score indicators of the two groups are "crying" and "response to operation". Crying should be an instinctive manifestation of newborns, but the frequency and degree of this index can affect the growth and development of newborns [14], in severe cases, necessary interventions are required. The scores of crying newborns in the KMC group are often lower than those of the control group. Similarly, in other pain indicators, the control group’s scores are generally higher than those of the KMC group. The intervention of pain indicators has a more positive effect. In the process of implementing kangaroo care, newborns are often relatively quiet. Studies have shown that kangaroo care can provide an environment similar to in-utero [15], giving newborns comfort and trust, which is safe The sensation may originate from the "pleasure" produced by the excitement of the limbic system during skin contact [16].

The influence of KMC on newborn health indicators In this study, newborns’ sleep time, vomiting frequency, crying time and frequency were selected as the observation indicators of neonatal health. During the research process, it was found that the number of sleep times and total sleep time in the KMC group were significantly longer than those in the control group. The analysis reasons were as follows: 1. KMC helps stabilize the newborn’s vital signs, keep the newborn’s sleep in a stable and comfortable state, which is conducive to long sleep 2. KMC gives newborns a sense of security, which makes it easier for newborns to fall asleep; 3. KMC can reduce the spontaneous or blind movement of the limbs caused by external stimuli after birth, and help to improve the quality of newborns’ sleep. 4. The transmission of mother's temperature, heartbeat, breathing, etc. helps to reduce the incompatibility of newborns to the outside world after birth, increase comfort and form a good sleep. Good sleep helps the development of the central nervous system and promotes the growth of newborns. development. Vomiting and overflowing milk are a common phenomenon in newborns. The common causes are that the newborn’s stomach volume is small, the structure and function development is not perfect, or the inhalation of more amniotic fluid causes swallowing syndrome. The increase in frequency and degree can lead to a series of Complications affect the health of newborns. This study found that after newborns were fed and burped, the vomiting rate of newborns who carried KMC was lower than that of newborns in the side lying state in the control group. Studies have reported that the effective alveolar ventilation of newborns in the prone position increases., The intestinal blood flow and gastrointestinal blood oxygen content increase, promote the development of neonatal gastrointestinal function. In the first half an hour after feeding, he was in the prone position, and the residual gastric volume was reduced, thereby reducing the feeding intolerance caused by the immature gastrointestinal function of the newborn [17]. Kangaroo care can also reduce neonatal nervousness, promote the secretion of neurotransmitters, regulate growth hormone, thyroxine and insulin levels, promote metabolism, and increase neonatal intake through early and continuous skin contact between mother and baby [18].

During the implementation of KMC, the number and time of crying in the KMC group was significantly less than that of the newborns in the control group. The reasons are the same as above, indicating that KMC has an effective role in comforting newborns. This topic also analyzed the weight changes of the two groups of newborns before they were discharged from the hospital. The weight gain of the newborns in the KMC group was higher than that of the control group. The reasons for the analysis are: 1. During the implementation of KMC, the premature infants relax and move independently. Reduced oxygen and calorie consumption is also conducive to weight gain; 2. KMC stabilizes various indicators of newborns and promotes metabolism; 3. KMC reduces the risk of newborn feeding intolerance, promotes intestinal absorption by newborns, and is beneficial to newborns Child growth and development.

The study on the effect of KMC on breastfeeding showed that the success rate of first breastfeeding and the rate of breastfeeding before discharge in the KMC group were higher than those of the control group. Among the pregnant women who had breastfeeding successfully for the first time, the average time to achieve success in the KMC group was shorter than that of the control group. Group, among the parturients who achieved exclusive breastfeeding before discharge, the KMC group achieved exclusive breastfeeding earlier than the control group. In the previous research of the research group, it was found that prolonging the skin contact time between mothers and infants after childbirth helped to increase the breastfeeding rate. This project encourages mothers to implement KMC as soon as possible after childbirth. Restrict people who conduct skin contact and extend the time of KMC as much as possible. The longest time for KMC to be implemented in this topic is 13.5 h per day. Long-term mother-to-child skin contact can help newborns to receive vomiting nipples and stimulate prolactin Secretion and prolong the time of hormone secretion, improve the efficiency of mother's milk production, enhance the mother's confidence in breastfeeding, and form a virtuous circle [19].

KMC is an important part of the WHO's early newborn care project. It plays an active role in promoting the growth and development of newborns, breastfeeding and other aspects. It is worthy of benefiting every newborn. At the same time, KMC is a simple and easy to master The neonatal care method shows strong and efficient advantages with its excellent clinical effects and low cost. During the period of poverty alleviation, health and poverty alleviation have been at the forefront. Ensuring the safety of mothers and babies is an important part of health and poverty alleviation. Liping County, Guizhou Province is the first ethnic minority area in China to carry out kangaroo care. The development of KMC has enabled local women and newborns to gain It has brought a good sense of experience and won unanimous praise from medical staff. It is a sustainable health care model that is worthy of further deepening and promotion.

For mothers and children, kangaroo mother care is not only suitable for premature babies but also for full-term babies. It can improve the rate of breastfeeding, stabilize the physiological condition of newborn babies, reduce crying, prolong sleep time and reduce the probability of infection. Skin-to-skin contact between mother and baby also reduces mother anxiety and enhances mother-baby emotional interaction. Kangaroo mother care gives babies a sense of security and intimacy, and is a great help in shaping their future personality.

On the social side, the guidance and companionship of perinatal professionals conform to nature. KMC's technology is simple and easy to master, and it does not need to consume large and high-value medical equipment, saving material costs. The main implementers of KMC are puerpera and their family members, which reduces the allocation of medical staff and saves the labor cost to a certain extent. The realization of exclusive breastfeeding can reduce the purchase of milk powder to the family brought economic pressure. Puerpera and their families can better understand medical staff and improve patient satisfaction, which is conducive to the establishment of a good doctor-patient relationship and social stability.

Meanwhile, there are some limitations of KMC technology. KMC originated in the period of lack of medical resources in Columbia and is a relatively primitive neonatal treatment method, which may not be recognized by some modern medical institutions and medical staff. Family members are required to fully understand and participate in practice. Any lack of these links may cause the interruption of KMC. There are certain requirements for room environment cleanliness and temperature and humidity (not suitable for areas where average temperature is too cold).

## Authors’ information

Wu Li,(1984-),female, gynecologist.Chongqing Health Center For Women And Children.Her main research direction is Pregnancy outcome of cervical insufficiency and neonatal care. Zhao Yu (1986-), male.Maternal and Child Health Hospital of Liping County Guizhou Province. deputy chief physician, his main research direction is the diagnosis and treatment of neonatal diseases and neonatal care.

## Data Availability

All original materials are agreed to be shared.The datasets used or analysed during the current study are available from the corresponding author on reasonable request.
